# Supplementary material for: Information transfer within and between autistic and non-autistic people
Source: Nat Hum Behav. 2025 May 14;9(7):1488–500. doi: 10.1038/s41562-025-02163-z (PMC12283400; doi:10.1038/s41562-025-02163-z)
Supplement: Supplementary file 1 — Tables 1.1 and 1.2 and descriptive statistics of the sample; linear and nonlinear models of task performance; post hoc models, which include additional predictor variables; and linear models of rapport. [file 41562_2025_2163_MOESM1_ESM.pdf]

nature human behaviour

Supplementary information https://doi.org/10.1038/s41562-025-02163-z

Information transfer within and between autistic and non-autistic people

In the format provided by the authors and unedited

Information transfer within and between autistic and non-autistic people

Supplementary Information

1. Descriptive Statistics 2

1.1 Demographics, IQ, and basic clinical information for autistic and non-autistic participants. 2

1.2 Demographics, IQ, and basic clinical information for participants by chain type (group) 4

2. Fictional Task Performance 6

2.1 Linear Models 6

2.2 Additional analyses to replicate Crompton et al., 2020. 6

3. Factual Task Performance 7

3.1 Linear Models 7

4. Non-linear approaches to Fictional and Factual analyses. 7

5. Post-hoc models including additional predictor variables (gender, IQ, ethnicity) for Fictional and Factual Story Task performance 8

5.1 Fictional Story 8

5.2 Factual Story 8

6. Self-rated rapport 9

6.1 Creating the Cumulative Rapport Score 9

6.2 Linear models of self-rated rapport 9

1. Descriptive Statistics

1.1 Demographics, IQ, and basic clinical information for autistic and non-autistic participants.

Table 1. Descriptive Statistics and Group Comparisons for Demographics, IQ, and Clinical Information by Diagnostic Status. Categorical variables were compared using Fisher's Exact Test, and continuous variables were compared using Wilcoxon Rank-Sum Test.

Characteristic	Autistic (n = 154)	Non-Autistic (n = 157)	p-value
**Age (years)**	28.68 ± 11.18	26.83 ± 11.26	.019
**Gender**			< .001
Woman	79 (51.30%)	118 (75.16%)	
Man	23 (14.94%)	35 (22.29%)	
Non-binary/gender neutral	47 (30.52%)	3 (1.91%)	
Prefer not to disclose	3 (1.95%)	1 (0.64%)	
Prefer to self-describe	2 (1.30%)	0	
**Highest Education Level Completed**			.698
Did not complete high school	4 (2.60%)	1 (0.64%)	
Completed high school	25 (16.23%)	29 (18.47%)	
Community college or vocational qualification	10 (6.49%)	8 (5.10%)	
Partially completed undergraduate degree	42 (27.27%)	43 (27.39%)	
Completed university undergraduate degree	32 (20.78%)	33 (21.02%)	
Partially completed postgraduate degree	20 (12.99%)	15 (9.55%)	
Completed postgraduate degree	21 (13.64%)	28 (17.83%)	
**Parental Education Level**			.056
Prefer not to say	3 (1.95%)	3 (1.91%)	
Did not complete high school	9 (5.84%)	6 (3.82%)	
Completed high school	23 (14.94%)	27 (17.20%)	
Community college or vocational qualification	14 (9.09%)	22 (14.01%)	
Partially completed undergraduate degree	13 (8.44%)	2 (1.27%)	
Completed university undergraduate degree	48 (31.17%)	51 (32.48%)	
Partially completed postgraduate degree	4 (2.60%)	1 (0.64%)	
Completed postgraduate degree	40 (25.97%)	45 (28.66%)	
**Ethnicity**			< .001
White	116 (75.32%)	87 (55.41%)	

Asian	14 (9.09%)	51 (32.48%)	
Black	8 (5.19%)	5 (3.18%)	
Mixed or Multiple Ethnicities	11 (7.14%)	8 (5.10%)	
Hispanic	3 (1.95%)	2 (1.27%)	
Other	2 (1.30%)	4 (2.55%)	
**IQ-WASI-II**[a]	115.62 ± 13.59	109.94 ± 11.49	< .001
**RAADS-14**[b]	31.02 ± 7.48	5.15 ± 4.17	
**Age of Diagnosis (years)**	23.72 ± 12.68	NA	

[a] IQ as assessed by the Wechsler Abbreviated Scale of Intelligence II (WASI-II)
[b] Autistic traits, as assessed by the Ritvo Autism and Asperger's Diagnostic Scale-Revised-14 item screen

1.2 Demographics, IQ, and basic clinical information for participants by chain type (group)

Table 2. Descriptive Statistics and Group Comparisons for Demographics, IQ, and Clinical Information. Means (standard deviations) for assigned groups are presented. Group comparisons were conducted using Kruskal–Wallis Chi-Square Test and Fisher's Exact Test.

Characteristic	Autistic (*n* = 103)	Mixed (*n* = 104)	Non-Autistic (*n* = 104)	p-value
**Age (years)**	29.35 ± 11.87	26.71 ± 9.52	27.18 ± 12.09	.877
**Gender**				<.001
Woman	52 (50.49%)	69 (66.35%)	76 (73.08%)	
Man	14 (13.59%)	17 (16.35%)	27 (25.96%)	
Non-binary/gender neutral	34 (33.01%)	16 (15.38%)	0	
Prefer not to disclose	2 (1.94%)	1 (0.96%)	1 (0.96%)	
Prefer to self-describe	1 (0.97%)	1 (0.96%)	0	
**Highest Education Level Completed**				.899
Did not complete high school	3 (2.91%)	1 (0.96%)	1 (0.96%)	
Completed high school	19 (18.45%)	14 (13.46%)	21 (20.19%)	
Community college or vocational qualification	4 (3.88%)	7 (6.73%)	7 (6.73%)	
Partially completed undergraduate degree	28 (27.18%)	29 (27.88%)	28 (26.88%)	
Completed university undergraduate degree	24 (23.30%)	20 (19.23%)	21 (20.19%)	
Partially completed postgraduate degree	12 (11.65%)	14 (13.46%)	9 (8.65%)	
Completed postgraduate degree	13 (12.62%)	19 (18.27%)	17 (16.35%)	
**Parental Education Level**				.078
Prefer not to say	1 (0.97%)	3 (2.88%)	2 (1.92)	
Did not complete high school	8 (7.77%)	2 (1.92%)	5 (4.81%)	
Completed high school	20 (19.42%)	8 (7.69%)	22 (21.15%)	
Community college or vocational qualification	9 (8.74%)	13 (12.50%)	14 (13.46%)	
Partially completed undergraduate degree	7 (6.73%)	7 (6.73%)	1 (0.96%)	
Completed university undergraduate degree	32 (31.07%)	36 (34.62%)	31 (29.81%)	
Partially completed postgraduate degree	2 (1.94%)	2 (1.92%)	1 (0.96%)	
Completed postgraduate degree	24 (23.30%)	33 (31.73%)	28 (26.92%)	

**Ethnicity**				< .001
White	81 (78.64%)	63 (60.58%)	59 (56.73%)	
Asian	8 (7.77%)	25 (24.04%)	32 (30.77%)	
Black	5 (4.85%)	3 (2.88%)	5 (4.81%)	
Mixed or Multiple Ethnicities	5 (4.85%)	9 (8.65%)	5 (4.81%)	
Hispanic	3 (2.91%)	0	2 (1.92%)	
Other	1 (0.97%)	4 (3.85%)	1 (0.96%)	
**IQ-WASI-IIa**	115.08 ± 14.28	114.76 ± 12.56	108.50 ± 10.56	< .001
**RAADS-14b**	35.11 ± 22.16	17.78 ± 13.70	4.78 ± 3.92	< .001
**Age of Diagnosis (years)**	24.24 ± 13.30	22.57 ± 11.24	NA	

[a] IQ as assessed by the Wechsler Abbreviated Scale of Intelligence
[b] Ritvo Autism and Asperger’s Diagnostic Scale-Revised 14 item screen

2. Fictional Task Performance

2.1 Linear Models

Linear Regression Modelling was used to examine the impact of multiple predictor variables on fictional information transfer. We first assessed linearity, normality, homoscedasticity, outliers, and multi-collinearity for fictional task performance. No strong violations of these five assumptions were observed.

A model comparison for fictional task performance in terms of AIC (Akaike Information Criterion) favoured a full model with all interactions, including the predictors *first* (fictional story first, factual story first), *site* (Dallas, Edinburgh, Nottingham) and diagnostic informing, or *info* for short (informed, uninformed). However, multi-collinearity in models with higher-order interactions suggested overfitting, so model ~ *chain type* * *order* + *site* * *info* * *first*[1] was selected as the most parsimonious model in terms of AIC and multi-collinearity (VIF<5). This model gave an adjusted R^2=0.54, indicating a significant improvement compared to an intercept-only model (F(16,294)=23.9, p<0.0001).

The factor c*hain type* had negligible effects (η^2<0.01) with non-significant differences of task performance between chain types. The effect of co-variate *order* was highly significant (b=-1.80, SE=0.23, t(294)=-7.82, p<0.0001, η^2=0.17). Control variable *site* had a small effect (η^2=0.02) with Edinburgh showing a significantly improved task performance compared to Dallas (b=2.10, SE=0.87, t(294)=2.41, p=0.02). Keeping participants uninformed about neurotypes in a chain as well as presenting the fictional story first had negligible effects on performance. There were significant two-way interactions between *site* Edinburgh and *first* Fictional (b=-5.63, SE=1.35, t(294)=-4.16, p<0.0001, η^2=0.06) and between *info* Uninformed and *first* Fictional (b=-5.03, SE=1.63, t(294)=-3.09, p=0.002, η^2=0.03) as well as a three-way interaction between site Edinburgh, info Uninformed and first Fictional (b=9.47, SE=2.22, t(294)=4.06, p<0.0001, η^2=0.05). No other main effects or interactions reached statistical significance.

In addition, the effect of *site* Edinburgh and fictional story *first* significantly reduced performance (b=-5.91, SE=2.35, t(54.1)=-2.52, p=0.015, η^2=0.07). The effect of *site* Edinburgh, *info* Uninformed and *first* Fictional significantly increased performance (b=9.85, SE=4.03, t(54.2)=2.44, p=0.018, η^2=0.06). No other effects reached statistical significance.

2.2 Additional analyses to replicate Crompton et al., 2020.

In an additional linear regression, we aimed to match the model and conditions used in the original study[34] as closely as possible. Thereto, we only analysed data from chains in which the fictional story was transferred first, and by participants who were informed about neurotype. Consequently, the sample was reduced from n=311 participants in 54 chains to n=92 participants in 16 chains. The results of the model ~ *chain type* * *order* indicated a small effect of *chain type* ($\eta^{2=}$0.01) with no significant differences between chain types but a large and significant effect of *order* (b=-1.97, SE=0.41, t(86) =-4.83, p<0.0001, η^2<0.21). The interaction effect between *chain type* and *order* was small ($\eta^{2=}$0.02) and revealed no significant differences.

---

[1] In the following all model specifications we follow the notation of R-package lme4[61]

3. Factual Task Performance

3.1 Linear Models

Linear Regression Modelling was used to examine the impact of multiple predictor variables on factual task performance. We first assessed linearity, normality, homoscedasticity, outliers, and multi-collinearity for factual task performance. No strong violations were noted.

A model comparison in terms of AIC favoured the full factorial model with interactions. However, the most complex models with higher-order interactions indicated multi-collinearity (VIF>5). As the most parsimonious model we therefore selected the model ~ *chain type* * *order* + *site* * *info* * *first*. Applying this model to factual task performance gave an adjusted R^2=0.58, indicating a significant better fit than the intercept-only model (F(16,294)=27.8, p<0.0001). The effects of *chain type* were small (η^2 =0.01) with non-significant differences between chain types. However, the effect of *order* was large with a highly significant reduction in task performance across participants in each chain (b=-1.55, SE=0.16, t(294)=-9.67, p<0.0001, η^2=0.24). Control variable *site* had a significant effect with Edinburgh showing a significantly improved task performance compared to Dallas (b=2.03, SE=0.61, t(294)=3.34, p=0.0009, η^2=0.04). Keeping participants uninformed about neurotypes in each chain significantly reduced performance (b=-1.55, SE=0.77, t(291)=-2.01, p=0.045, η^2=0.01). Presenting the fictional story rather than factual story *first* also had a small effect and increased factual task performance significantly (b=1.63, SE=0.66, t(294)=2.45, p=0.015, η^2=0.02).

There was a significant effect of *site* Edinburgh and *first* Fictional (b=-4.21, SE=0.94, t(294)=-4.47, p<0.0001, η^2=0.06) as well as for *site* Nottingham, *info* Uninformed and *first* Fictional (b=-5.70, SE=1.59, t(294)=-3.59, p=0.0004, η^2=0.04). No other effects reached statistical significance.

The performance for *site* Edinburgh was significantly better than for Dallas (b=2.46, SE=1.04, t(40.2)=2.37, p=0.023, η^2=0.05). There was also a significant effect of *site* Edinburgh and *first* Fictional (b=-4.55, SE=1.35, t(53.8)=-3.37, p<0.001, η^2=0.08). No other effects reached statistical significance.

4. Non-linear approaches to Fictional and Factual analyses.

In an alternative approach, we also implemented a non-linear multi-level mixed-effect model with two parameters and exponential decay across participants (R-package brms[64]) separately for fictional and factual task performance. The non-linear model b_1* *exp*(b_2 * *order*) had random intercepts for chain number b_1 ~ 1 + (1 | number). Rate of information decay was modelled by a linear combination of fixed effects plus random intercepts for chain number b_2 ~ *chain type* + *site* + *info* + *first* + (1 | number). All model parameters converged well (R-hat< 1.01) and results confirmed the absence of any significant effects involving the factor *chain type*. However, this non-linear model with exponential decay did not perform significantly better in terms of leave-one-out information criterion looic (R-package loo[65,66]) than the most parsimonious MLM model with *log(order)* as a covariate. Therefore, the results of the analyses are not included here, but are available in our post-hoc analysis files.

5. Post-hoc models including additional predictor variables (gender, IQ, ethnicity) for Fictional and Factual Story Task performance

Linear Mixed Effects Modelling was used to examine the impact of multiple predictor variables on fictional information transfer, including the covariates of gender, IQ, and ethnicity. Here we present analogous models to the ones in the main paper, with the addition of the covariates (the model ~ *chain type* * *log(order)* + *site* * *info* * *first* + (1 + *log(order)* | number). The aim is to explore how these covariates, alongside core predictors like chain type and order, influence information retention and decay within different chain types (autistic, non-autistic, and mixed). We first assessed linearity, normality, homoscedasticity, outliers, and multi-collinearity for fictional task performance. No strong violations of these five assumptions were observed.

5.1 Fictional Story

This model predicted task performance (range 0-30) with an adjusted (marginal) R^2=0.56 and adjusted (conditional) R^2=0.86 (R-package MuMIn[56]). Satterthwaite's method was used to adjust degrees of freedom in *t*-tests (R-package lmerTest[57]).

As predicted, the main effect of log(order) was large and statistically significant (b=-5.13, SE=0.56, t(52.3)=-9.10, p<0.0001, η^2=0.18, r2glmm package). There was no significant difference in task performance across chain types (autistic, non-autistic, and mixed). However, a significant interaction between chain type Autistic and log(order) showed that task performance was reduced more in the Autistic group compared to the Non-autistic group (b=-1.74, SE=0.80, t(50.9)=-2.19, p=0.033, η^2=0.01). IQ was also a significant predictor, with higher IQ scores associated with higher task performance (b=0.04, SE=0.01, t(220.9)=3.71, p<0.001, η^2=0.06).

5.2 Factual Story

This model predicted task performance (range 0-30) with an adjusted (marginal) R^2=0.62 and adjusted (conditional) R^2=0.89 (MuMIn package). Satterthwaite's method was used to adjust degrees of freedom in t-tests (lmerTest package).

As expected, the main effect of log(order) was large and statistically significant (b=-4.46, SE=0.53, t(54.3)=-8.42, p<0.0001, η^2=0.27, r2glmm package). Site effects were significant, with participants from Edinburgh performing significantly better than those from Dallas (b=2.66, SE=0.88, t(50.8)=3.01, p=0.004, η^2=0.07).

First-person fictional accounts were also significant predictors, where task performance increased when participants were exposed to fictional information (b=2.47, SE=0.96, t(49.3)=2.56, p=0.014, η^2=0.12). IQ had a significant positive effect on task performance (b=0.03, SE=0.01, t(218.6)=3.84, p<0.001, η^2=0.06).

6. Self-rated rapport

6.1 Creating the Cumulative Rapport Score

As a measure of scale reliability, we applied Cronbach's alpha[67] (R-package psych[68]) to the five subscales of the rapport score ("successful", "easy", "enjoyable", "friendly", and reverse-coded "awkward"). The results indicate values greater than 0.8 which is above the acceptable threshold of 0.7 for teacher/learner rapport as well as fictional/factual stories. Therefore, all subscales were included in the accumulated rapport score (ranging from 0 to 500).

6.2 Linear models of self-rated rapport

Linear regression models were used to examine the impact of multiple predictor variables on teacher and learner rapport scores. We assessed linearity, normality, homoscedasticity, outliers, and collinearity for teacher and learner rapport scores separately; no strong violations were noted.

A model comparison for teacher rapport in terms of AIC favoured a linear model where ~ *chain type* + *story_type* * *first* + *info* + *site* predicted the teacher rapport scores, with factor *story type* discriminating between the rapport scores from the fictional and factual story. Including higher-order interactions between the five factors made only marginal contributions but increased multi-collinearity (VIF>5). This model gave an adjusted R^2=0.06, indicating a significant improvement compared to an intercept-only model ($F(8,505)=5.11$, $p<0.0001$).

The effects of factor *chain type* suggest a significantly lower teacher rapport score for mixed chains (b=-36.0, SE=9.12, $t(505)$=-3.95, p=0.0001, η^2=0.03) as well as autistic chains (b=-33.49, SE=9.12, $t(505)$=-3.67, p=0.0003, η^2=0.03) compared to non-autistic chains. The effect of *site* Nottingham had a significant lower rapport score than Dallas (b=-20.58, SE=9.02, $t(505)$=-2.28, p=0.023, η^2=0.01) whereas Edinburgh was not significantly lower than Dallas (b=-8.80, SE=9.17, $t(505)$=-0.96, p=0.338). The effect of diagnostic informing or *info* suggests that teachers who were uninformed about the neurotype of the learner had a significantly lower rapport score than teachers who were informed (b=-17.95, SE=7.91, $t(505)$=-2.27, p=0.024, η^2=0.01). There was an effect of *first* indicating that fictional story *first* significantly reduced the overall rapport scores (b=-71.88, SE=23.65, $t(505)$=-3.04, p=0.003, η^2=0.02). The effect of *story type* was negligible but the interaction effect between *story type* Factual and *first* Fictional was significant (b=36.52, SE=14.93, $t(505)$=2.45, p=0.015, η^2=0.01). No other statistically significant effects were observed.

An equivalent model predicted the learner rapport score with an adjusted R^2=0.07 ($F(8,503)$=5.86, $p<0.0001$). The effects of *chain type* gave a statistically significant difference for mixed chains (b=-22.6, SE=8.25, $t(503)$=-2.74, p=0.006, η^2=0.02) as well as autistic chains (b=-20.5, SE=8.21, $t(503)$=-2.49, p=0.013, η^2=0.01) compared to non-autistic chains. The *site* Nottingham had a significant lower rapport score than Dallas (b=-17.70, SE=8.15, $t(503)$=-2.17, p=0.03, η^2=0.01) whereas Edinburgh was not significantly different from Dallas (b=-13.94, SE=8.27, $t(503)$=-1.69, p=0.093, η^2=0.01). According to *diagnostic informing* (*info)* learners who were uninformed about the neurotype of the teacher had a significantly lower rapport score than learners who were informed (b=-20.23, SE=7.10, $t(503)$=-2.85, p=0.005, η^2=0.02). There was an effect of *first* indicating that transfer of the fictional story first significantly reduced the overall rapport scores (b=-45.3, SE=9.56, $t(503)$=-4.74, $p<0.0001$, η^2=0.04). The effect of *story type* was not significant but the effect for *story type* Factual and *first* Fictional was highly significant (b=50.2, SE=13.46, $t(503)$=3.73, p=0.0002, η^2=0.03). No other statistically significant effects were observed.
