# Supplementary material for: Information transfer within and between autistic and non-autistic people
Source: Nat Hum Behav. 2025 May 14;9(7):1488–500. doi: 10.1038/s41562-025-02163-z (PMC12283400; doi:10.1038/s41562-025-02163-z)
Supplement: Supplementary file 2 — Reporting Summary [file 41562_2025_2163_MOESM2_ESM.pdf]

nature portfolio

Corresponding author(s): Dr Catherine Crompton.
Last updated by author(s): 2025- 01- 28

# Reporting Summary

Nature Portfolio wishes to improve the reproducibility of the work that we publish. This form provides structure for consistency and transparency in reporting. For further information on Nature Portfolio policies, see our Editorial Policies and the Editorial Policy Checklist.

## Statistics

For all statistical analyses, confirm that the following items are present in the figure legend, table legend, main text, or Methods section.

| n/a | Confirmed | |
|---|---|---|
| ☐ | ☒ | The exact sample size (*n*) for each experimental group/condition, given as a discrete number and unit of measurement |
| ☐ | ☒ | A statement on whether measurements were taken from distinct samples or whether the same sample was measured repeatedly |
| ☐ | ☒ | The statistical test(s) used AND whether they are one- or two-sided<br>*Only common tests should be described solely by name; describe more complex techniques in the Methods section.* |
| ☐ | ☒ | A description of all covariates tested |
| ☐ | ☒ | A description of any assumptions or corrections, such as tests of normality and adjustment for multiple comparisons |
| ☐ | ☒ | A full description of the statistical parameters including central tendency (e.g. means) or other basic estimates (e.g. regression coefficient) AND variation (e.g. standard deviation) or associated estimates of uncertainty (e.g. confidence intervals) |
| ☐ | ☒ | For null hypothesis testing, the test statistic (e.g. *F*, *t*, *r*) with confidence intervals, effect sizes, degrees of freedom and *P* value noted<br>*Give P values as exact values whenever suitable.* |
| ☐ | ☒ | For Bayesian analysis, information on the choice of priors and Markov chain Monte Carlo settings |
| ☐ | ☒ | For hierarchical and complex designs, identification of the appropriate level for tests and full reporting of outcomes |
| ☐ | ☒ | Estimates of effect sizes (e.g. Cohen's *d*, Pearson's *r*), indicating how they were calculated |

*Our web collection on statistics for biologists contains articles on many of the points above.*

## Software and code

Policy information about availability of computer code

| | |
|---|---|
| Data collection | No software was used during data collection |
| Data analysis | All code for analysis is freely and openly available on the Open Science Framework page for this study (https://osf.io/us9c7/) |

For manuscripts utilizing custom algorithms or software that are central to the research but not yet described in published literature, software must be made available to editors and reviewers. We strongly encourage code deposition in a community repository (e.g. GitHub). See the Nature Portfolio guidelines for submitting code & software for further information.

Analyses were conducted in R version 4.4.2, using packages lme4: Version 1.1.35.5; bayesfactor: Version 0.9.12.4.7; MuMIn: Version 1.48.4; r2glmm: Version 0.1.2; lmerTest: Version 3.1.3; simr: Version 1.0.7; brms: Version 2.22.0; nlme: Version 3.1.166; fpp2: Version 2.5; rstan: Version 2.32.6

## Data

Policy information about availability of data

All manuscripts must include a data availability statement. This statement should provide the following information, where applicable:

- Accession codes, unique identifiers, or web links for publicly available datasets
- A description of any restrictions on data availability
- For clinical datasets or third party data, please ensure that the statement adheres to our policy

All data are freely and openly available on the Open Science Framework page for this study (https://osf.io/us9c7/)

# Research involving human participants, their data, or biological material

Policy information about studies with human participants or human data. See also policy information about sex, gender (identity/presentation), and sexual orientation and race, ethnicity and racism.

| | |
|---|---|
| Reporting on sex and gender | We attempted to recruit a sample that was representative of a range of genders, and attempted to ensure that the autistic and non-autistic groups were comparable on gender.<br><br>Participants self-reported their gender on an initial demographic questionnaire. Of the 311 participants, 197 (63.34%) were women, 58 (18.65%) were men, 50 (16.07%) were non-binary, and 6 (1.94%) preferred not to disclose their gender or self-identified as another gender. Of the autistic participants specifically (n = 154), 79 (51.30%) were women, 23 (14.94%) were men, 47 (30.52%) were non-binary, and 5 (3.25%) preferred not to disclose or self-identified as another gender. Of the non-autistic participants specifically (n = 157), 118 (75.16%) were women, 35 (22.29%) were men, 3 (1.91%) were non-binary, and 1 (0.64%) preferred not to disclose or self-identified as another gender. A breakdown of participant genders by the chain condition (autistic, non-autistic, and mixed) is available in the Supplement.<br><br>We do not anticipate any differences on performance based on gender. However, as the groups significantly differed on gender, gender was included as an additional control variable in post-hoc analysis, but did not affect the results. This is described in the Supplement. |
| Reporting on race, ethnicity, or other socially relevant groupings | We attempted to recruit a sample that was representative of a range of ethnicities, and attempted to ensure that the autistic and non-autistic groups were comparable on ethnicity.<br><br>Participants self-reported their ethnicity on an initial demographic questionnaire. These questionnaires used census-based frameworks for each of the countries where data were collected (USA, England, Scotland) and were then collapsed into higher-level categories to enhance comparability.<br><br>Of the 311 participants, 203 (65.27%) were White, 65 (20.90%) were Asian, 19 (6.11%) were Mixed or Multiple Ethnicities, 13 (4.18%) were Black, 5 (1.61%) were Hispanic, and 6 (1.93%) identified as 'Other'. Of the autistic participants specifically (n = 154), 116 (75.32%) were White, 14 (9.09%) were Asian, 11 (7.14%) were Mixed or Multiple Ethnicities, 8 (5.19%) were Black, 3 (1.95%) were Hispanic, and 2 (1.30%) identified as 'Other'. Of the non-autistic participants specifically (n = 157), 87 (55.41%) were White, 51 (32.48%) were Asian, 8 (5.10%) were Mixed or Multiple Ethnicities, 5 (3.18%) were Black, 2 (1.27%) were Hispanic, and 4 (2.55%) identified as 'Other'. A breakdown of participant ethnicities by the chain condition (autistic, non-autistic, and mixed) is available in the Supplement.<br><br>We do not anticipate any differences on performance based on ethnicity. However, as the groups significantly differed on ethnicity, ethnicity was included as an additional control variable in post-hoc analysis, but did not affect the results. This is described in the Supplement. |
| Population characteristics | We attempted to recruit a sample that was representative of a range of ages, and attempted to ensure that the autistic and non-autistic groups were comparable on age. Autistic participants had a mean age of 28.68 (SD = 11.18) and non-autistic participants had a mean age of 26.83 (SD = 11.26). A breakdown of participant age by the chain condition (autistic, non-autistic, and mixed) is available in the Supplement.<br><br>Participants provided details of the highest educational level achieved during their initial demographic questionnaire, and both groups were matched on educational level (see Supplement for full details).<br><br>Of the 311 participants, 5 (1.61%) had not completed high school, 54 (17.36%) had completed high school, 18 (5.79%) completed community college or vocational qualifications, 158 (50.80%) had a partially completed or completed undergraduate degree, and 84 (27.00%) had a partially completed or completed postgraduate degree. Of the autistic participants specifically (n = 154), 4 (2.60%) had not completed high school, 25 (16.23%) had completed high school, 10 (6.49%) completed community college or vocational qualifications, 74 (48.05%) had a partially completed or completed undergraduate degree, and 41 (26.63%) had a partially completed or completed postgraduate degree. Of the non-autistic participants specifically (n = 157), 1 (0.64%) had not completed high school, 29 (18.47%) had completed high school, 8 (5.10%) completed community college or vocational qualifications, 76 (48.41%) had a partially completed or completed undergraduate degree, and 43 (27.39%) had a partially completed or completed postgraduate degree. Details of the educational levels for autistic participants and non-autistic participants presented separately are available in the Supplement, along with a breakdown of participant education level by the chain condition (autistic, non-autistic, and mixed).<br><br>Participant IQ was measured using the Wechsler Abbreviated Scale of Intelligence-II. Autistic participants had a mean IQ of 118.14 (SD = 15.50), and non-autistic participants had a mean IQ of 111.46 (SD = 12.95). A breakdown of group-level IQ scores by the chain condition (autistic, non-autistic, and mixed) is available in the Supplement.<br><br>The autistic group included participants reporting a clinical diagnosis of autism (n = 144) and those who self-identify as being autistic (n = 40). Autistic participants mean age of diagnosis (or self-diagnosis) was 23.72 years (SD = 12.68). Participants who self-identifed as autistic completed the Ritvo Autism and Asperger's Diagnostic Scale-Revised (RAADS-R)48 and were included if their score was above 72, as recommended in the literature. All participants completed the Ritvo Autism and Asperger's Diagnostic Scale 14-item Screen (RAADS-14). Autistic participants had a mean score of 33.39 (SD = 18.59); non-autistic participants had a mean score of 5.15 (SD = 4.17). A breakdown of RAADS scores and age-of diagnosis by chain conditions that included autistic (autistic, mixed)) is available in the Supplement. |

Recruitment

324 adult participants were recruited across three sites (University of Edinburgh, University of Nottingham, and the University of Texas at Dallas) and 311 (154 autistic, 157 non-autistic) attended research days. Participants were recruited through databases held at each University (Edinburgh: the Patrick Wild Centre Participant Database; Nottingham: the Autism Research Team Database; Dallas: The Autism Research Collaborative), partnerships with local autism charities and autistic organisations, and social media. Due to the recruitment being largely via Universities, it is likely that our sample is relatively highly educated, which may impact results. This point is included in the Discussion.

Ethics oversight

This study was carried out in accordance with the British Psychological Society's Code on Human Research Ethics and the American Psychological Association's Ethical Principles of Psychologists and Code of Conduct. Experimental procedures were reviewed and approved by the University of Edinburgh's Medical Research Ethics Committee, University of Nottingham, School of Psychology Ethics Committee, and the University of Texas at Dallas's Institutional Review Board. All participants provided written informed consent before participating and were remunerated for their time (£30/$40).

Note that full information on the approval of the study protocol must also be provided in the manuscript.

# Field-specific reporting

Please select the one below that is the best fit for your research. If you are not sure, read the appropriate sections before making your selection.

☐ Life sciences ☒ Behavioural & social sciences ☐ Ecological, evolutionary & environmental sciences

For a reference copy of the document with all sections, see nature.com/documents/nr-reporting-summary-flat.pdf

# Behavioural & social sciences study design

All studies must disclose on these points even when the disclosure is negative.

Study description

This study used a mixed experimental design incorporating both between and within-groups factors. Between-groups factors included chain type (autistic, non-autistic, mixed) and diagnostic-informing (informed, uninformed), with task type (fictional, factual) as a within-group factor.

Diffusion chain tasks and the Rapport task generated quantitative data (see Data Collection below for full details).

Research sample

Autistic and non-autistic participants were older than 18 years, of any gender, spoke English equivalent to a native level, and had normal/corrected normal sight and hearing. Participants were ineligible if they had a diagnosis of Social Anxiety Disorder or uncontrolled epilepsy. All participants provided written informed consent before participating and were remunerated for their time .

All participants completed the Ritvo Autism and Asperger's Diagnostic Scale 14-item Screen (RAADS-14), and non-autistic participants were excluded from participating if their scores indicated high levels of autistic traits (score > 14).

We aimed to recruit a diverse, representative sample including a range of genders, ages, ethnicities, and intellectual abilities. Our sample does have a fairly high level of education and IQ, and so results should be interpreted with this in mind.

Sampling strategy

Participants were recruited using convenience sampling.

Sample size was determined using an a-priori power analysis outlined in our Stage 1 submission, and is detailed below.

Expected effect sizes: There are few scientific comparisons between autistic, non-autistic, and mixed social groups. The analysis in the original study gave a partial h2 effect size of 0.45 for chain type, 0.83 for position, and 0.08 for the interaction of chain type and position, though there are insufficient similar studies to know if these are reliable effect sizes. We, therefore, propose being conservative in our effect size estimates given the paucity of data, especially in high-powered studies. In the proposed study we suggest a sample size that is based on a revised data analysis and powered to detect medium effects in the data.

Power analysis: A mixed design for chains with between and within factors appears more appropriate than a between-subjects design. This increases the power of the study. We modified the linear model so that each chain (rather than participant) is treated as an independent observation and proportion of recalled information from a participant in a chain is considered as a repeated measure, allowing for dependencies between participants within a chain. Applying a corresponding linear model with repeated measurements to the original data by Crompton et al. (2020) suggests larger effect sizes for the main effects and interaction (chain type partial h2 =0.52, position in chain 0.87, interaction 0.20). For equivalent analyses of rapport scores we found a partial h2 of 0.19 for the interaction.)

Since the main effects are strong, the smallest meaningful interaction effect between chain type and position would be a medium effect of h2 =0.06 (partial Cohen's f =0.25). In order to establish a correlation coefficient for the within-factor position we have to introduce assumptions about the correlation matrix. For compound symmetry we estimated r =0.502 using a general least square fit (function gls() in R-package nlme. (Fitting an auto-regressive AR(1) correlation matrix to the data increased the coefficient to ϕ =0.767 but this fit was not significantly better than the compound symmetry fit.) Assuming a statistical significance level of p=0.05, a medium effect size of h2 =0.06, a lower correlation of r=0.4 and a correction for non-sphericity of e =0.7 (Greenhouse-Geisser) then a power analysis59 for a within-between interaction in an ANOVA with repeated measures 54 chains with 6 positions (participants)

suggests a total of 324 participants to reach 95% power (see also Figure 1 at https://osf.io/us9c7/ ).

A priori power analyses for linear mixed-effect models are notoriously difficult to conduct and require simulation studies. A simulation-based power analysis requires fitting a linear mixed model to the existing data by Crompton et al., with 8 participants in each of 3 chains for each condition (N=72). Since this data set has the minimum number of chains per condition we can only fit a mixed model with random intercepts. If 'position' is added as a further random effect then the model fails to converge and we can no longer run a simulation-based power analysis.

We calculated the exact power for the interaction effect of a mixed-effect linear model with a random intercept for each chain using the R-packages lme4 and simr. Based on the estimated coefficients of the mixed-effect model analysis on the original data (omitting the data for chain position 7 and 8) Monte-Carlo simulations gave power estimates for different numbers of chains. The simulation results are conservative because they are based on the estimated coefficients reduced by one third. The simulations suggest more than 45 chains to test the fixed effect of interaction chain type by position with 95% power (see Figure 2 at https://osf.io/us9c7/).

Further details of the power analysis and simulations in R can be found in the R-file PowerAnalysis at https://osf.io/us9c7/

The large sample of 324 participants is the maximum feasible under current funding constraints and should provide sufficient power (>95%) to test the hypotheses and to explore undirected and two-way interaction effects in post-hoc analyses. For the main hypotheses we will also compute Bayes factors for normally distributed differences between means (R-package BayesFactor). Unlike Neyman-Pearson statistical inference Bayes factors accumulate evidence with increasing sample size and inform about the likelihood of the alternative relative to the null hypothesis given the evidence. We will also conduct comprehensive model comparisons using information criteria to establish the most parsimonious (mixed-effect/non-linear) model.

Our proposed sample (324 participants in 54 chains) is considerably larger than the original sample (72 participants in 9 chains) in Crompton et al. There are several benefits of this. First, we are powered (>95%) to detect and replicate results for reduced effect sizes. Second, this sample size should also give us the opportunity to fit the maximal model and/or to identify the most parsimonious model – rather than relying on the intercept-only model used in the simulation. Third, it enables us to examine further undirected effects, namely comparing across informed and uninformed conditions, sites, and content type with effect sizes that are likely to be smaller. Finally, it allows us to account for potential data loss or outliers. The sample size is therefore substantially larger than the original study, and far exceeds the sample size of previous studies reporting similar group differences. The increased sample and therefore number of chains is further justified because variability within chains can be investigated using not only linear but also non-linear and dynamic models (see post-hoc analyses).

Data collection

Chain types: This study involved comparing performance on information transfer and rapport scores between three groups(chain types) to which participants were assigned upon enrolment. The chain types were: autistic chains, where all participants are autistic; non-autistic chains, where all participants are non-autistic, and mixed chains, where half the participants are autistic and half the participants are non-autistic.

Revealing diagnostic status: This study examined information transfer in two conditions; (1) the informed condition, where participants were aware of the diagnostic status of the participants in their diffusion chain group and (2) the uninformed condition, where participants were not informed of the diagnostic status of the other participants in their diffusion type. 105 participants were allocated to the uninformed condition, and 206 participants were allocated to the informed condition. This facilitated a direct replication of the original study (where participants were informed about diagnostic status) with a sample size powered to detect smaller effects, while also permitting assessment of the effect of diagnostic-information on information transfer within the resources available for this study. The distribution of participants to these conditions was because of the funding constraints of this study. Specifically, we applied for funding to a scheme designed solely to fund replications and powered our study on that basis. In response to reviewer comments the funder offered additional support to allow us to extend the study to include a smaller, uninformed diagnosis condition, and this resulted in the imbalanced groups.

Content type: This study examined whether the efficacy of information transfer differs for factual and fictional information. Content type was counterbalanced across the chains, with half of all chains first completing a fictional task, and half first completing the factual task.

Procedure
The experimental diffusion chain tasks: This study used a diffusion chain methodology - a controlled, experimental form of the game "Telephone" - which has been effective in probing cultural learning between individuals in a social group52,53. In this method, an experimenter models a complex behaviour to the first person in the chain; in this case, verbally telling the participant a short passage of text. The person then has a chance to rehearse re-telling the information alone before being paired with the next person in the chain and instructed tell them the passage. After hearing the passage, the second participant can practice the behaviour and then must pass it on to the next individual, and so on. Before commencing a diffusion chain, we ensured that consecutive participants in the chain did not know one another.

In practice, this meant that the researcher played a video to the first participant (A) where a non-autistic man read the passage aloud. The researcher then left the room and a second participant (B) entered. Participant A then recounted the story to Participant B. A then left the room, and a third participant (C) entered. Participant B then recounted the story to Participant C and so on, to the sixth participant (F). The sixth participant recounted the story aloud, alone. Participants waited in separate rooms for their turn, to avoid contamination during the information sharing. For mixed chains, half began with an autistic participant and half began with a non-autistic participant before alternating between autistic and non-autistic participants. All diffusion chains were video recorded for scoring purposes.

In each diffusion chain six participants completed two separate diffusion chain tasks: a fictional and factual task. In each chain six participants completed one task in full – for instance passing a fictional story through all six people in the chain - and then the same chain in the same participant order completed the second task. The order of fictional and factual task administration (within group factor) was counterbalanced across chain types so that familiarity effects (from interacting with the same person twice) were

distributed evenly between fictional and factual task conditions.

The fictional condition task was a short story which was surreal and difficult to predict. The factual condition task involved a short passage describing facts of an obscure scientific nature. The passages for both tasks feature 30 individual details, allowing the task to be scored out of a maximum of 30 for each participant. Both tasks had comparable Flesch-Kincaid Grade Level and Flesch Reading Ease scores, featured a comparable number of words, and had comparable word and sentence lengths54,55. Both were designed to be completely novel to participants, difficult to predict and not involve any inherently social features.

A participant's final score corresponded to the number of details they recalled when recounting the passage to the next person in the chain, out of a maximum of 30. A higher score indicated a greater amount of information shared. Both passages and their scoring schemes are available on the study OSF website (https://osf.io/us9c7/), though were embargoed while data collection was underway to ensure that participants were unfamiliar with the content of the passages when they participated in the study. Two researchers independently coded 50% of the videos. Additionally, they each second coded 5% of randomly selected video material assigned to the other researcher, giving a 10% overlap of videos that were double coded. Inter-rater reliability was calculated using a Single Rating Absolute-Agreement 2-way Mixed-effects model as per56, and was very high for both tasks (factual ICC 0.986 (p<.0001), 95% CI [0.975, 0.992]; fictional ICC .978 (p<.0001), 95% CI [0.961, 0.987]).

Participants within each of the chains were ordered in ascending age, to minimise a possible effect of age-related memory decline. Chains were also organised to minimise frequent switches of gender in order to avoid a possible effect on information transfer and rapport.

Participants in the informed condition were told whether they were in an autistic, non-autistic, or mixed chain. Participants in the uninformed condition were not informed about chain types. Participants did not meet before the study started and waited in separate rooms throughout the study, except when participating in the diffusion chains.

The experimental rapport measure: Participants were asked to rate the rapport they experienced while completing the diffusion chain tasks. Participants completed these rapport measures twice: once for the interaction when they were the 'listener' (i.e., when they were listening to another participant recount the passage to them) and once for the interaction when they were the 'speaker' (i.e., when they were recounting the passage to another participant). The first participant in each chain only rated rapport as a speaker, and the last participant in the chain only rated rapport as a listener.

The rapport measure used was taken from the original Crompton et al. study34,40,41 and involved participants answering using a slider on a scale from 0 to 100 (1) how much did you enjoy the interaction? (2) how easy was the interaction? (3) how successful was the interaction? (4) how friendly was the interaction? (5) how awkward was the interaction? (reverse scored). The full measure can be found on the OSF website (https://osf.io/us9c7/).

Studies of rapport in dyadic interactions typically use self-rated questionnaires57. While self-rated rapport may be subject to response biases (for example, if autistic people underestimate their rapport due to negative self-perception of social skills or a history of difficult interactions with others; or if non-autistic people overestimate their rapport40, we consider that it is nevertheless the optimal way to assess each participants' direct experience of the interaction.

Specifically, self-rated rapport was selected over observer-rated rapport, as most methods developed for measuring observer-rated rapport do not accommodate neurodiverse interactional experiences. External rapport measures can be biased by a neuro-normative lens: normative external indicators of rapport are less likely to be observed between autistic pairs40, and this may be undetected or misinterpreted by observers. This means that even if autistic pairs are experiencing high rapport, external observers are likely to rate them as having low rapport.

For example, when independent observers rate videos of autistic people, they rate them as being more awkward and less approachable14, both of which are key factors in building rapport. These biases are robust, are developed very rapidly, and do not change with increased exposure14. Importantly, autistic independent observers have a similar tendency to non-autistic judges to evaluate autistic adults less favourably than non-autistic adults in videos16, and so this bias cannot be overcome by simply recruiting both autistic and non-autistic independent judges. Similarly, emotion recognition is subject to strong neuro-normative biases – autistic people have different facial expressions to non-autistic people, and non-autistic people (and thus, emotion recognition software based on non-autistic norms) are poor at identifying these emotions28, which is related to their perceiving them unfavourably. Normative biases have also been detected within intelligent learning algorithms58, and thus automated tools based on machine learning are similarly problematic in this context.

Additionally, since we examined whether rapport varies depending on social context (single or mixed dyad), rather than as a main effect of diagnosis (autistic or non-autistic), any influence of response bias associated with autism was well-managed by the study design. For these reasons, self-rated rapport was used in this study.

Standardised tasks: To characterise the IQ of the sample and match across groups, participants completed the Wechsler Abbreviated Scale of Intelligence II (WASI-II) two-subtest version59. All participants completed the Ritvo Autism and Asperger's Diagnostic Scale 14-item Screen (RAADS-14)50 to characterise the sample; additionally, participants who self-identify as autistic completed the Ritvo Autism and Asperger's Diagnostic Scale-Revised (RAADS-R)48.

Protocol: Participants completed the tasks in the following order.

Online – in advance of in person participation
- Information sheet and consent form
- Demographic questionnaire
- Ritvo Autism and Asperger's Diagnostic Scale (Ritvo et al., 2011) / Ritvo Autism and Asperger's Diagnostic Scale 14-item Screen (RAADS-14; Eriksson et al., 2013)
-
In person participation
- WASI – II (Wechsler, 2011)

- Diffusion Chain Task 1
- Rapport Measure 1
- Diffusion Chain Task 2
- Rapport Measure 2

The Stage 1 protocol for this Registered Report was accepted in principle on 23rd August 2022. The protocol, as accepted by the journal, can be found at https://osf.io/us9c7/

Researcher knowledge: Researchers were aware of the diagnostic status of all participants, and thus data collection, scoring, and analysis was not performed blind to the conditions of the experiment.

Timing

Participants were recruited between August 2022 and October 2023.

Data exclusions

Three research days produced data of insufficient quality or quantity. Thus, the research from these days were not included, and the diffusion chains re-run with new participants. Our reasons for excluding these data were (1) one day only had four participants attend, so there was not sufficient data to include (2) one chain included a participant in position one who recalled a very low level of information, below the outlier threshold of ±2.5 standard deviations from the mean outlined in our Sampling Plan, and (3) we had an unequal balance of missing data from five-person chains across the three conditions (autistic, non-autistic, mixed); in order to ensure that missing data was balanced across the three conditions, an additional autistic chain was re-run with six participants.

Non-participation

We aimed to recruit 324 participants (18 chains consisting of 6 people in each of the autistic, non-autistic, and mixed conditions). Due to a small number of participants (n =13) not attending, 13 chains contained only five participants (5 autistic, 4 non-autistic, 4 mixed). No reason was given by participants for their non-attendance.

Our power analysis suggests that our planned analyses are robust for up to 5.5% missing values; with our actual missing data of 4% below this threshold.

Randomization

This study used non-randomised samples, and participants were assigned to either a non-autistic, autistic, or a mixed autistic-non-autistic chain and to the informed or uninformed condition according to diagnostic status, gender, order of recruitment, and participant availability.

The order of fictional task first of factual task first was counterbalanced across the diffusion chains, and accounted for in the analysis by its inclusion as a predictor variable

# Reporting for specific materials, systems and methods

We require information from authors about some types of materials, experimental systems and methods used in many studies. Here, indicate whether each material, system or method listed is relevant to your study. If you are not sure if a list item applies to your research, read the appropriate section before selecting a response.

## Materials & experimental systems

| n/a | Involved in the study |
|---|---|
| x | ☐ Antibodies |
| x | ☐ Eukaryotic cell lines |
| x | ☐ Palaeontology and archaeology ☐ |
| X | ☐ Animals and other organisms |
| X | ☐ Clinical data |
| X | ☐ Dual use research of concern |
| X | ☐ Plants |

## Methods

| n/a | Involved in the study |
|---|---|
| X | ☐ ChIP-seq |
| X | ☐ Flow cytometry |
| X | ☐ MRI-based neuroimaging |

## Antibodies

Antibodies used

*Describe all antibodies used in the study; as applicable, provide supplier name, catalog number, clone name, and lot number.*

Validation

*Describe the validation of each primary antibody for the species and application, noting any validation statements on the manufacturer's website, relevant citations, antibody profiles in online databases, or data provided in the manuscript.*

## Eukaryotic cell lines

Policy information about cell lines and Sex and Gender in Research

Cell line source(s)

*State the source of each cell line used and the sex of all primary cell lines and cells derived from human participants or vertebrate models.*

Authentication

*Describe the authentication procedures for each cell line used OR declare that none of the cell lines used were authenticated.*

| | |
|---|---|
| Mycoplasma contamination | *Confirm that all cell lines tested negative for mycoplasma contamination OR describe the results of the testing for mycoplasma contamination OR declare that the cell lines were not tested for mycoplasma contamination.* |
| Commonly misidentified lines (See ICLAC register) | *Name any commonly misidentified cell lines used in the study and provide a rationale for their use.* |

## Palaeontology and Archaeology

| | |
|---|---|
| Specimen provenance | *Provide provenance information for specimens and describe permits that were obtained for the work (including the name of the issuing authority, the date of issue, and any identifying information). Permits should encompass collection and, where applicable, export.* |
| Specimen deposition | *Indicate where the specimens have been deposited to permit free access by other researchers.* |
| Dating methods | *If new dates are provided, describe how they were obtained (e.g. collection, storage, sample pretreatment and measurement), where they were obtained (i.e. lab name), the calibration program and the protocol for quality assurance OR state that no new dates are provided.* |

☐ Tick this box to confirm that the raw and calibrated dates are available in the paper or in Supplementary Information.

| | |
|---|---|
| Ethics oversight | *Identify the organization(s) that approved or provided guidance on the study protocol, OR state that no ethical approval or guidance was required and explain why not.* |

Note that full information on the approval of the study protocol must also be provided in the manuscript.

## Animals and other research organisms

Policy information about studies involving animals; ARRIVE guidelines recommended for reporting animal research, and Sex and Gender in Research

| | |
|---|---|
| Laboratory animals | *For laboratory animals, report species, strain and age OR state that the study did not involve laboratory animals.* |
| Wild animals | *Provide details on animals observed in or captured in the field; report species and age where possible. Describe how animals were caught and transported and what happened to captive animals after the study (if killed, explain why and describe method; if released, say where and when) OR state that the study did not involve wild animals.* |
| Reporting on sex | *Indicate if findings apply to only one sex; describe whether sex was considered in study design, methods used for assigning sex. Provide data disaggregated for sex where this information has been collected in the source data as appropriate; provide overall numbers in this Reporting Summary. Please state if this information has not been collected. Report sex-based analyses where performed, justify reasons for lack of sex-based analysis.* |
| Field-collected samples | *For laboratory work with field-collected samples, describe all relevant parameters such as housing, maintenance, temperature, photoperiod and end-of-experiment protocol OR state that the study did not involve samples collected from the field.* |
| Ethics oversight | *Identify the organization(s) that approved or provided guidance on the study protocol, OR state that no ethical approval or guidance was required and explain why not.* |

Note that full information on the approval of the study protocol must also be provided in the manuscript.

## Clinical data

Policy information about clinical studies

All manuscripts should comply with the ICMJE guidelines for publication of clinical research and a completed CONSORT checklist must be included with all submissions.

| | |
|---|---|
| Clinical trial registration | *Provide the trial registration number from ClinicalTrials.gov or an equivalent agency.* |
| Study protocol | *Note where the full trial protocol can be accessed OR if not available, explain why.* |
| Data collection | *Describe the settings and locales of data collection, noting the time periods of recruitment and data collection.* |
| Outcomes | *Describe how you pre-defined primary and secondary outcome measures and how you assessed these measures.* |

## Dual use research of concern

Policy information about dual use research of concern

### Hazards

Could the accidental, deliberate or reckless misuse of agents or technologies generated in the work, or the application of information presented in the manuscript, pose a threat to:

| No | Yes | |
|---|---|---|
| ☐ | | Public health |
| ☐ | | National security |
| ☐ | | Crops and/or livestock |
| ☐ | | Ecosystems |
| ☐ | | Any other significant area |

## Experiments of concern

Does the work involve any of these experiments of concern:

| No | Yes | |
|---|---|---|
| ☐ | | Demonstrate how to render a vaccine ineffective |
| ☐ | | Confer resistance to therapeutically useful antibiotics or antiviral agents |
| ☐ | | Enhance the virulence of a pathogen or render a nonpathogen virulent |
| ☐ | | Increase transmissibility of a pathogen |
| ☐ | | Alter the host range of a pathogen |
| ☐ | | Enable evasion of diagnostic/detection modalities |
| ☐ | | Enable the weaponization of a biological agent or toxin |
| ☐ | | Any other potentially harmful combination of experiments and agents |

# Plants

| | |
|---|---|
| Seed stocks | *Report on the source of all seed stocks or other plant material used. If applicable, state the seed stock centre and catalogue number. If plant specimens were collected from the field, describe the collection location, date and sampling procedures.* |
| Novel plant genotypes | *Describe the methods by which all novel plant genotypes were produced. This includes those generated by transgenic approaches, gene editing, chemical/radiation-based mutagenesis and hybridization. For transgenic lines, describe the transformation method, the number of independent lines analyzed and the generation upon which experiments were performed. For gene-edited lines, describe the editor used, the endogenous sequence targeted for editing, the targeting guide RNA sequence (if applicable) and how the editor was applied.* |
| Authentication | *Describe anyau thentication procedures for each seedstocku sed or novel genotype generated. Describe any experiments usedto assess the effect of a mutation and, where applicable, how potential secondary effects (e.g. second site T-DNA insertions, mosiacism, off-target gene editing) were examined.* |

# ChIP-seq

## Data deposition

☐ Confirm that both raw and final processed data have been deposited in a public database such as GEO.

☐ Confirm that you have deposited or provided access to graph files (e.g. BED files) for the called peaks.

| | |
|---|---|
| Data access links<br>*May remain private before publication.* | *For "Initial submission" or "Revised version" documents, provide reviewer access links. For your "Final submission" document, provide a link to the deposited data.* |
| Files in database submission | *Provide a list of all files available in the database submission.* |
| Genome browser session<br>(e.g. UCSC) | *Provide a link to an anonymized genome browser session for "Initial submission" and "Revised version" documents only, to enable peer review. Write "no longer applicable" for "Final submission" documents.* |

## Methodology

| | |
|---|---|
| Replicates | *Describe the experimental replicates, specifying number, type and replicate agreement.* |
| Sequencing depth | *Describe the sequencing depth for each experiment, providing the total number of reads, uniquely mapped reads, length of reads and whether they were paired- or single-end.* |
| Antibodies | *Describe the antibodies used for the ChIP-seq experiments; as applicable, provide supplier name, catalog number, clone name, and lot number.* |

Peak calling parameters | *Specify the command line program and parameters used for read mapping and peak calling, including the ChIP, control and index files used.*

Data quality | *Describe the methods used to ensure data quality in full detail, including how many peaks are at FDR 5% and above 5-fold enrichment.*

Software | *Describe the software used to collect and analyze the ChIP-seq data. For custom code that has been deposited into a community repository, provide accession details.*

# Flow Cytometry

## Plots

Confirm that:

☐ The axis labels state the marker and fluorochrome used (e.g. CD4-FITC).

☐ The axis scales are clearly visible. Include numbers along axes only for bottom left plot of group (a 'group' is an analysis of identical markers).

☐ All plots are contour plots with outliers or pseudocolor plots.

☐ A numerical value for number of cells or percentage (with statistics) is provided.

## Methodology

Sample preparation | *Describe the sample preparation, detailing the biological source of the cells and any tissue processing steps used.*

Instrument | *Identify the instrument used for data collection, specifying make and model number.*

Software | *Describe the software used to collect and analyze the flow cytometry data. For custom code that has been deposited into a community repository, provide accession details.*

Cell population abundance | *Describe the abundance of the relevant cell populations within post-sort fractions, providing details on the purity of the samples and how it was determined.*

Gating strategy | *Describe the gating strategy used for all relevant experiments, specifying the preliminary FSC/SSC gates of the starting cell population, indicating where boundaries between "positive" and "negative" staining cell populations are defined.*

☐ Tick this box to confirm that a figure exemplifying the gating strategy is provided in the Supplementary Information.

# Magnetic resonance imaging

## Experimental design

Design type | *Indicate task or resting state; event-related or block design.*

Design specifications | *Specify the number of blocks, trials or experimental units per session and/or subject, and specify the length of each trial or block (if trials are blocked) and interval between trials.*

Behavioral performance measures | *State number and/or type of variables recorded (e.g. correct button press, response time) and what statistics were used to establish that the subjects were performing the task as expected (e.g. mean, range, and/or standard deviation across subjects).*

## Acquisition

Imaging type(s) | *Specify: functional, structural, diffusion, perfusion.*

Field strength | *Specify in Tesla*

Sequence & imaging parameters | *Specify the pulse sequence type (gradient echo, spin echo, etc.), imaging type (EPI, spiral, etc.), field of view, matrix size, slice thickness, orientation and TE/TR/flip angle.*

Area of acquisition | *State whether a whole brain scan was used OR define the area of acquisition, describing how the region was determined.*

Diffusion MRI ☐ Used ☐ Not used

## Preprocessing

Preprocessing software | *Provide detail on software version and revision number and on specific parameters (model/functions, brain extraction, segmentation, smoothing kernel size, etc.).*

| | |
|---|---|
| Normalization | *If data were normalized/standardized, describe the approach(es): specify linear or non-linear and define image types used for transformation OR indicate that data were not normalized and explain rationale for lack of normalization.* |
| Normalization template | *Describe the template used for normalization/transformation, specifying subject space or group standardized space (e.g. original Talairach, MNI305, ICBM152) OR indicate that the data were not normalized.* |
| Noise and artifact removal | *Describe your procedure(s) for artifact and structured noise removal, specifying motion parameters, tissue signals and physiological signals (heart rate, respiration).* |
| Volume censoring | *Define your software and/or method and criteria for volume censoring, and state the extent of such censoring.* |

## Statistical modeling & inference

| | |
|---|---|
| Model type and settings | *Specify type (mass univariate, multivariate, RSA, predictive, etc.) and describe essential details of the model at the first and second levels (e.g. fixed, random or mixed effects; drift or auto-correlation).* |
| Effect(s) tested | *Define precise effect in terms of the task or stimulus conditions instead of psychological concepts and indicate whether ANOVA or factorial designs were used.* |

Specify type of analysis: ☐ Whole brain ☐ ROI-based ☐ Both

| | |
|---|---|
| Statistic type for inference<br>(See Eklund et al. 2016) | *Specify voxel-wise or cluster-wise and report all relevant parameters for cluster-wise methods.* |
| Correction | *Describe the type of correction and how it is obtained for multiple comparisons (e.g. FWE, FDR, permutation or Monte Carlo).* |

## Models & analysis

| n/a | Involved in the study |
|---|---|
| ☐ | ☐ Functional and/or effective connectivity |
| ☐ | ☐ Graph analysis |
| ☐ | ☐ Multivariate modeling or predictive analysis |

| | |
|---|---|
| Functional and/or effective connectivity | *Report the measures of dependence used and the model details (e.g. Pearson correlation, partial correlation, mutual information).* |
| Graph analysis | *Report the dependent variable and connectivity measure, specifying weighted graph or binarized graph, subject- or group-level, and the global and/or node summaries used (e.g. clustering coefficient, efficiency, etc.).* |
| Multivariate modeling and predictive analysis | *Specify independent variables, features extraction and dimension reduction, model, training and evaluation metrics.* |
